# *Schistosoma japonicum* HSP60-derived peptide SJMHE1 suppresses delayed-type hypersensitivity in a murine model

**DOI:** 10.1186/s13071-016-1434-4

**Published:** 2016-03-12

**Authors:** Xuefeng Wang, Jun Wang, Yong Liang, Hongchang Ni, Liang Shi, Chengcheng Xu, Yuepeng Zhou, Yuting Su, Xiao Mou, Deyu Chen, Chaoming Mao

**Affiliations:** Department of Central Laboratory, The Affiliated Hospital of Jiangsu University, Zhenjiang, 212001 China; Department of Nuclear Medicine and Institute of Oncology, The Affiliated Hospital of Jiangsu University, Zhenjiang, 212001 China; Department of Nuclear Medicine, The Affiliated People’s Hospital of Jiangsu University, Zhenjiang, Jiangsu 212002 China; Clinical Laboratory, Huai’an Hospital Affiliated of Xuzhou Medical College, Huaian, Jiangsu 223300 China

**Keywords:** *Schistosoma japonicum*-derived peptide, SJMHE1, Suppress, Delayed-type hypersensitivity

## Abstract

**Background:**

Parasite-derived molecules with immunomodulatory properties, which have been optimised during host-parasite co-evolution, exhibit potential applications as novel immunotherapeutics. We have previously demonstrated that *Schistosoma japonicum* HSP60-derived peptide SJMHE1 induces CD4^+^CD25^+^ regulatory T-cells (Tregs) and that adoptively transferred SJMHE1-induced CD4^+^CD25^+^ Tregs inhibit delayed-type hypersensitivity (DTH) in mice. However, multiple concerns regarding this method render this treatment unsuitable. To gain further insights into the potential effects of SJMHE1, we used ovalbumin (OVA)-induced DTH and evaluated the effect of SJMHE1 on DTH mice.

**Methods:**

BALB/c mice were sensitised with OVA alone or combined with SJMHE1 and then challenged with OVA to induce DTH. We first analysed the potential effects of SJMHE1 by measuring DTH responses, T-cell responses, cytokine secretion, and Treg proportions. We then evaluated the expression levels of IL-10 and TGF-β1 in CD4^+^CD25^+^ T-cells during DTH and Treg generation to identify the mechanism by which SJMHE1 suppresses DTH.

**Results:**

SJMHE1 modulated the effector response against OVA-induced DTH and stimulated the production of the anti-inflammatory cytokines IL-10 and TGF-β1 in immunised mice through a mechanism involving CD4^+^CD25^+^ Tregs. SJMHE1-induced CD4^+^CD25^+^ Tregs expressed high levels of CTLA-4, IL-10, and TGF-β1, which substantially contributed to the suppressive activity during DTH. The administration of SJMHE1 to DTH in mice led to the expansion of CD4^+^CD25^+^ Tregs from CD4^+^CD25^−^ T-cells in the periphery, which inhibited DTH responses.

**Conclusions:**

Our study proves that the parasite-driven peptide suppresses DTH in mice, which may confer a new option for inflammation treatment.

## Background

Helminth infections exert potent systemic immunomodulatory effects on the host immune system, weakening host response to both infectious and noninfectious antigens [1, 2]. The capacity of helminth parasites to modulate the immune system underpins their longevity in the mammalian host [3–5]. The remarkable range of parasite life histories, transmission strategies, and physiological niches is reflected in the variety of immunomodulatory activities observed [3, 6, 7]. For instance, schistosome infections lead to antigen-specific unresponsiveness in the peripheral T-cell populations of heavily infected patients [8, 9]. Moreover, concurrent helminth infection decreases the response to bystander allergens and autoantigens in both model systems and human studies [1, 10, 11]. Thus, a comparison of the mechanisms of laboratory-based rodent-helminth model system with clinical assessment of individuals infected with helminth parasites could reveal ways to manipulate the human immune system to treat auto-immune and inflammatory diseases. This process has been clinically implemented as patients with inflammatory bowel diseases or allergic diseases are being deliberately infected with parasitic worms to evaluate their therapeutic use. Existing findings clearly indicate that infection with helminth parasites can reduce the severity of these diseases [12–14].

Instead of infecting people with pathogens, which predisposes them to the inevitable risk of side effects, a more responsible approach is to identify the immunomodulatory molecules that selectively mimic the desirable effects of infection and use them as a novel therapeutic approach [6, 15, 16]. Data from animal models (and to a lesser extent, human studies) show that helminths release products that interfere with the development of allergic responses and inflammatory diseases [11, 17, 18]. Considerable studies have focused on identifying novel products that exhibit similar properties. Beneficial products are expected to be identified, characterised, and tested *in vivo* in the near future.

Schistosomiasis is a typical helminth infection that induces immunomodulation [19, 20]. Infection with schistosomes or exposure to schistosome-derived antigens prevents the occurrence of various auto-immune disorders and atopic diseases [21–23]. Mechanistically, molecules produced by a schistosome at different stages of its life-cycle in the mammalian host can potentially inhibit both auto-immune and inflammatory diseases through various mechanisms [19]. We identified an HSP60-derived peptide SJMHE1 from *Schistosoma japonicum* and demonstrated that SJMHE1 stimulates IL-10 and TGF-β, as well as inhibits IL-12 and TNF-α production by macrophages and dendritic cells, leading to the development of CD4^+^CD25^+^ Tregs. Using an adoptive transfer model, we further demonstrated that SJMHE1 inhibits DTH by inducing CD4^+^CD25^+^ Tregs [24]. However, isolation of peptide-induced Treg populations requires highly specialised facilities, and the procedure can entail high costs [25]. Thus, immunotherapy based on the peptide induction of Tregs may have limited therapeutic potential.

We investigated the potential effects of SJMHE1 on ovalbumin (OVA)-induced DTH to develop the medical potential of the therapeutic peptide and to elucidate the mechanism by which SJMHE1-induced CD4^+^CD25^+^ cells downregulate DTH responses. Results showed that SJMHE1 modulated the effector response against OVA-induced DTH and induced the production of the anti-inflammatory cytokines IL-10 and TGF-β1 in mice sensitised with OVA combined with SJMHE1. The modulation of the immune response to OVA by SJMHE1 resulted from the induction of CD4^+^CD25^+^ Tregs. The administration of SJMHE1 to DTH mice led to the expansion of CD4^+^CD25^+^ Tregs from CD4^+^CD25^−^ T-cells in the periphery, which inhibited DTH responses. These findings may provide useful information for exploring the potential therapeutic application of parasite-derived molecules.

## Methods

### Ethics statement

Animal experiments were performed in strict accordance with the Regulations for the Administration of Affairs Concerning Experimental Animals (1988.11.1), and efforts were exerted to minimise the suffering of the animals. All animal procedures were approved by the Institutional Animal Care and Use Committee (IACUC) of Jiangsu University for the use of laboratory animals (Permit Number: JSU 13-027).

### Mice

Eight-week-old female BALB/c mice were purchased from the SLAC Laboratory (Shanghai, China). All animal experiments were conducted in accordance with the Chinese laws for animal protection and with the experimental guidelines and procedures approved by the IACUC of Jiangsu University for the use of laboratory animals.

### Peptides

SjHSP60 437-460 (SJMHE1) (VPGGGTALLRCIPVLDTLSTKNED) was synthesised and purified by Top-peptide (Shanghai, China). The purity of the peptides was determined to be greater than 99 % by mass spectrometry. SJMHE1 was pretreated with polymyxin B-agarose in accordance with a previously described method [26] to exclude possible LPS contamination.

### DTH induction and assessment

Each mouse was primed in the rear footpad with 100 μg of OVA (fraction V; Sigma, Poole, UK) alone or combined with 10, 20, or 30 μg of SJMHE1 emulsified with complete Freund’s adjuvant (Sigma) in 100 μL. The control group received 100 μL of equal mixtures of PBS and CFA. Seven days after sensitisation, the mice were challenged with the subcutaneous injection of 20 μL of OVA (1 mg/mL in PBS) in the left ear and 20 μL of PBS in the right ear. The dosage and volume of OVA for sensitisation and challenge were based on previous studies [24, 27]. DTH was assessed by measuring the thickness of the challenged ear before and 24 h after the challenge in a blind manner with the use of a micrometer (Mitutoyo, Osaka, Japan).

Mice were sacrificed 24 h post-challenge, and their ears were removed. The ear tissues were homogenised for cytokine measurement.

For CD4^+^CD25^+^ T-cell depletion, BALB/c mice were treated intraperitoneally with 500 μg of the anti-CD25 monoclonal antibody clone PC61 (BD Bioscience, Pharmingen, San Diego, CA, USA) 24 h before immunisation with OVA as previously described [28]. Depletion efficiency was verified by flow cytometry (FCM) as previously described [29].

### Cell isolation

Single-cell suspensions were prepared from the pooled lymph nodes (LNs) and spleens of six mice per group in RPMI 1640 containing 10 % FCS. CD4^+^CD25^+^ and CD4^+^CD25^−^ cell populations were separated using a mouse Treg isolation kit (Miltenyi Biotec, Auburn, CA, USA) in accordance with the manufacturer’s instructions. The purity of the resulting CD4^+^CD25^+^ and CD4^+^CD25^−^ populations was routinely 95–98 %, as determined by FCM. APCs were obtained and irradiated from single-cell suspensions in accordance with a previously described method [24].

### Cell culture

For the proliferation assay, one day after OVA challenge, cell suspensions were generated from the pooled LN and spleens from individual mice as described above. Cells were incubated in RPMI-1640 containing 10 % FCS, 2 mM L-glutamine, 100 U/mL penicillin, 100 mg/mL streptomycin, and 1.25 mg/mL amphotericin B (all Gibco BRL, CA, USA) (complete medium) in the presence of 1, 10, and 100 mg/mL OVA at 37 °C in 5 % CO_2_. Cell proliferation was evaluated by [^3^H] thymidine (^3^H-TdR) incorporation. Cytokine content was analysed in culture supernatants by ELISA from Bender Med Systems, Vienna, Austria.

For suppression assays, 1 × 10^5^ CD4^+^CD25^−^ T-cells/well, 5 × 10^4^ CD4^+^CD25^+^ T-cells/well, or both populations were cultured in 96-well U-bottom plates with 1 × 10^5^ APCs/well for 72 h at 37 °C in complete RPMI 1640 medium (0.2 mL/well) in triplicate. Cultures were stimulated with 1 μg/mL soluble anti-CD3 (BD PharMingen, San Diego, CA, USA) with or without 0.1 μg/mL SJMHE1. Certain wells were added with 3 μg/mL rat IgG1 anti-mouse IL-10 (Biolegend Inc., San Diego, CA, USA), 0.5 μg/mL rat IgG1 anti-mouse TGF-β1 (US Biological, Swampscott, MA, USA), or 3 μg/mL rat IgG1 (Biolegend). Proliferation was assessed by incubation with 0.5 μCi/well ^3^H-thymidine and measuring the incorporation during the final 16 h of culture.

### Cytokine quantitation

TNF-α, IL-12, IL-10, and TGF-β1 in the supernatants of splenic lymphocyte stimulated by 100 μg/mL OVA or in the supernatants of homogenised ear were analysed using an ELISA kit (Bender Med Systems, Vienna, Austria) in accordance with the manufacturer’s instructions.

### Flow cytometry

The Mouse Regulatory T-Cell Staining Kit (eBioscience, San Diego, CA, USA) was used. To analyse CD4^+^CD25^+^Foxp3^+^ or CD4^+^CD25^+^CTLA4^+^ T-cells, splenic and LN cells were pooled from the mice treated with PBS, sensitised to OVA alone, or sensitised to OVA and 30 μg of SJMHE1. They were surface-stained with PerCP anti-CD3 mAbs (eBioscience, San Diego, CA, USA), FITC anti-CD4 mAbs, APC anti-CD25 mAbs, and PE anti-CTLA4. Certain cells were fixed, and then permeabilised with Cytofix/Cytoperm. Finally, they were stained intracellularly with phycoerythrin (PE) mouse anti-Foxp3 or PE IgG2a rat immunoglobulin control antibody in accordance with the manufacturer’s instructions.

To detect intracellular cytokines, splenic and LN cells from mice that were treated with PBS, sensitised to OVA alone, or sensitised to OVA and 30 μg of SJMHE1 were stimulated in the presence of PMA (25 ng/mL), ionomycin (1 μg/mL), and GolgiStop™ (0.66 μL/mL) at 2 × 10^6^/mL (2 mL/well) in 24-well plates for 6 h at 37 °C in 5 % CO_2_. After incubation with anti-CD3-PerCP, anti-CD4-FITC, and anti-CD25-APC mAbs, the cells were washed, fixed, and then permeabilised with Cytofix/Cytoperm solution (BD PharMingen). The cells were stained intracellularly with PE-conjugated anti-IL-10 mAb (0.2 mg/mL), anti-TGF-β1 mAb (0.5 mg/mL), or rat IgG1 (isotype control) for 1 h at room temperature. Finally, the cells were washed in FACS buffer (PBS, 2 % FCS and 0.05 % sodium azide) and then analysed with the FACS Calibur (Becton Dickinson, San Jose, CA) by using the CellQuest software (BD Biosciences).

### Statistical analysis

Statistical analysis was performed using GraphPad Prism 5.01 (GraphPad Software, 2007, La Jolla, CA, USA). Statistical significance was determined using Student’s *t*-test at the *P* < 0.05 level.

## Results

### SHMHE1 suppressed DTH responses and modulated local cytokine secretion

**Fig. 1:**
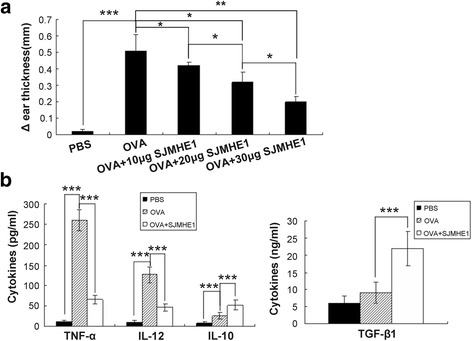
Suppression of DTH responses by SJMHE1. **a** BALB/c mice were sensitised with OVA alone or combined with various amounts of SJMHE1 (as indicated). Challenge with OVA occurred 7 days later, and the DTH responses were assessed over the subsequent 24 h with the change in ear thickness. The DTH responses are expressed as the mean ± SD of 12 mice from two independent experiments; **b** BALB/c mice were sensitised with OVA alone or combined with 30 μg of SJMHE1. Challenge with OVA occurred 7 days later; 24 h after the challenge, the ear was removed and homogenised. Cytokine levels in the supernatants were measured from the homogenised tissue. Data are shown as the mean ± SD of 12 mice from two independent experiments. Asterisks indicate significant differences analysed using Student’s *t*-test (*P < 0.05; **P < 0.01; ***P < 0.001)

To examine whether or not SJMHE1 can modulate DTH responses against the unrelated protein OVA, BALB/c mice were sensitised in the rear footpad with OVA alone or combined with SJMHE1 emulsified in complete Freund’s adjuvant. After seven days, each mouse was challenged in the left ear with OVA. Monitoring of the subsequent swelling in the challenged ear showed that sensitisation and challenge with OVA resulted in the pronounced thickening of the ear, which signified DTH response. Sensitisation in the presence of SJMHE1 significantly suppressed DTH responses. This effect was dose dependent, and 30 μg of SJMHE1 induced a strong effect to prevent the development of DTH (Fig. 1a).

Local cytokine production in the DTH ears was measured to analyse further the *in vivo* immune suppression induced by SJMHE1. The ears were removed and homogenised 24 h after the challenge; multiple cytokines were then measured. Considering that 30 μg of SJMHE1 induced the strongest inhibition of DTH response, we investigated local cytokine production in the DTH ears from mice primed with OVA alone or combined with 30 μg of SJMHE1. The cytokine levels in the control ears from PBS mice were undetectable (Fig. 1b). By contrast, elevated levels of pro-inflammatory cytokines (TNF-α and IL-12) were detected in the DTH ear primed and challenged with OVA alone. Priming in the presence of SJMHE1 significantly inhibited the local production of TNF-α (*t* = 25.09, *P* < 0.001) and IL-12 (*t* = 12.64, *P* < 0.001) but induced high levels of the anti-inflammatory cytokines IL-10 (*t* = 6.485, *P* < 0.001) and TGF-β1 (*t* = 7.723, *P* < 0.001) (Fig. 1b). These results suggest that SJMHE1 can suppress DTH, reduce local pro-inflammatory cytokines, and increase local anti-inflammatory cytokines.

### SJMHE1 modulated OVA-specific T-cell responses and cytokine secretion in DTH mice

**Fig. 2:**
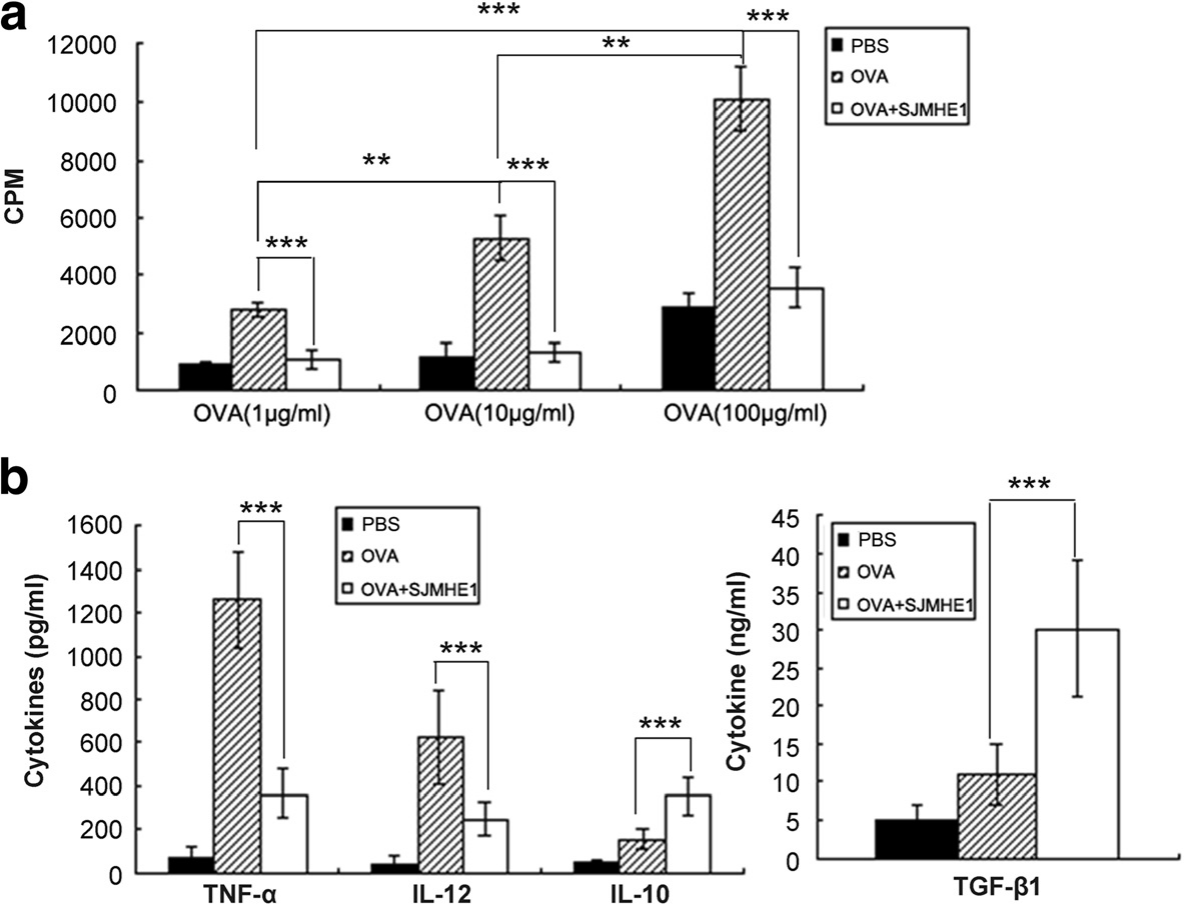
SJMHE1 modulated OVA-specific T-cell responses and cytokine secretion in DTH mice. **a** BALB/c mice were sensitised with OVA alone or combined with 30 μg of SJMHE1. Challenge with OVA occurred 7 days later, and pooled splenic and LN cells from these mice were prepared 1 day after the challenge. The cells were cultured in complete RPMI 1640 at 5 × 10^5^ cells/well with OVA for 3 days, and proliferation was measured by ^3^H-thymidine incorporation. Data are expressed as the mean values of two experiments with six mice per group; **b** Cells were cultured at 5 × 10^5^ cells/well stimulated by 100 μg/mL OVA for 3 days, and the cytokines in culture supernatants were analysed by ELISA. Data are displayed as the mean ± SD from two experiments performed in triplicate. Asterisks indicate significant differences analysed using the independent Student’s *t*-test (**P < 0.01; ***P < 0.001)

Examination of local pro-inflammatory cytokine production strongly suggested that exposure to SJMHE1 *in vivo* altered the effector properties of OVA-specific T-cells. Therefore, OVA-specific T-cell proliferation and cytokine production were examined after stimulation by OVA *ex vivo* to characterise further the functional phenotype of OVA-specific T-cells from mice that were primed and challenged by OVA alone or combined with SJMHE1. As shown in Fig. 2a, splenic lymphocytes from mice primed with only OVA showed a dose-dependent proliferation profile. However, the splenic lymphocytes from mice primed with OVA combined with 30 μg of SJMHE1 exhibited significantly reduced proliferative capacity after OVA stimulation *ex vivo* (1 μg/mL OVA stimulation: *t* = 18.57, *P* < 0.001; 10 μg/mL OVA stimulation: *t* = 15.78, *P* < 0.001; 100 μg/mL OVA stimulation: *t* = 17.78, *P* < 0.001; Fig. 2a). Therefore, OVA combined with SJMHE1 elicited a more observable OVA-specific T-cell unresponsiveness compared with the other treatments.

Splenic lymphocytes were cultured *ex vivo* with 100 μg/mL OVA to evaluate the effects of SJMHE1 treatment on cytokine secretion by T-cells. The levels of TNF-α, IL-12, IL-10, and TGF-β1 were measured in the culture supernatant through ELISA. As shown in Fig. 2b, the splenic lymphocytes from mice primed with only OVA secreted large amounts of TNF-α and IL-12 in response to OVA but produced minimal IL-10 and TGF-β1. Meanwhile, the splenic lymphocytes from mice primed with OVA combined with SJMHE1 produced high levels of IL-10 (*t* = 6.749, *P* < 0.001) and TGF-β1 (*t* = 6.725, *P* < 0.001) but decreased levels of TNF-α (*t* = 12.38, *P* < 0.001) and IL-12 (*t* = 5.631, *P* < 0.001). These findings suggest that SJMHE1 induces anti-inflammatory cytokines (IL-10 and TGF-β1) to protect against DTH. Overall, SJMHE1 induces an OVA-specific T-cell unresponsiveness and anti-inflammatory environment to weaken the pro-inflammatory response and thus protect against DTH.

### SJMHE1 increased CD4^+^CD25^+^ Foxp3^+^ Treg proportions in DTH mice

Previous reports suggested that soluble mediators such as IL-10 and TGF-β1 contribute to the induction of CD4^+^CD25^+^ Tregs [29, 30], which consequently secrete IL-10 and/or TGF-β1 to enhance the inhibition of DTH responses [25, 29]. SJMHE1 induces the *ex vivo* production of regulatory cytokines IL-10 and TGF-β1 in DTH mice, and SJMHE1 treatment increases CD4^+^CD25^+^ Tregs both *in vivo* and *in vitro* [24]. Thus, we assumed that the inhibition of DTH responses in mice treated with SJMHE1 is potentially associated with CD4^+^CD25^+^ Tregs induced by SJMHE1. We then tested the CD4^+^CD25^+^FoxP3^+^ T-cells from mice treated with OVA alone or combined with SJMHE1.

**Fig. 3:**
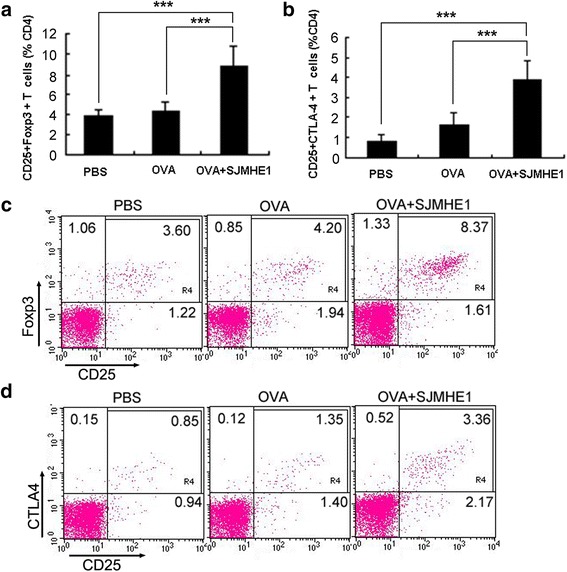
SJMHE1 increased CD4^+^CD25^+^ Foxp3^+^ T-cells in DTH mice. BALB/c mice were sensitised with OVA alone or combined with 30 μg of SJMHE1. Challenge with OVA occurred 7 days later; 24 h after the challenge, spleen and LNs from each mouse were pooled. Single-cell suspensions were prepared, and red blood cells were lysed. **a** Flow cytometry for CD3, CD4, CD25, and Foxp3 was performed, and data are expressed as the mean ± SD of 18 mice from three independent experiments; **b** Flow cytometry for CD3, CD4, CD25, and CTLA4 was performed. Data are expressed as the mean ± SD of 18 mice from three independent experiments; **c** Analysis of CD4^+^CD25^+^Foxp3^+^ T-cells from pooled splenic and LN cells by flow cytometry. Data are representative of the experiments; **d** Analysis of CD4^+^CD25^+^CTLA4^+^ T-cells from pooled spleen and LN cells by flow cytometry. Data are representative of the experiments. Values indicate the percentage of events in the indicated quadrant. Asterisks indicate significant differences analysed using the independent Student’s *t*-test (***P < 0.001)

As shown in Fig. 3a and c, the proportion of CD4^+^CD25^+^FoxP3^+^ T-cells significantly increased in the spleens and LNs of the mice sensitised with OVA combined with SJMHE1 compared with those of the mice sensitised with OVA alone (*t* = 8.785, *P* < 0.001) or treated with PBS (*t* = 10.17, *P* < 0.001). SJMHE1 treatment upregulated the expression of a regulatory characteristic marker (cytotoxic T lymphocyte antigen 4, CTLA-4) on CD4^+^CD25^+^ T-cells (OVA *vs* OVA + SJMHE1: *t* = 8.404, *P* < 0.001; PBS *vs* OVA + SJMHE1: *t* = 12.52, *P* < 0.001; Fig. 3b and d). Overall, SJMHE1 promotes the generation of activated CD4^+^CD25^+^ Tregs during DTH.

### SJMHE1 induced IL-10 and TGF-β1 expression in CD4^+^CD25^+^ T-cells during DTH

**Fig. 4:**
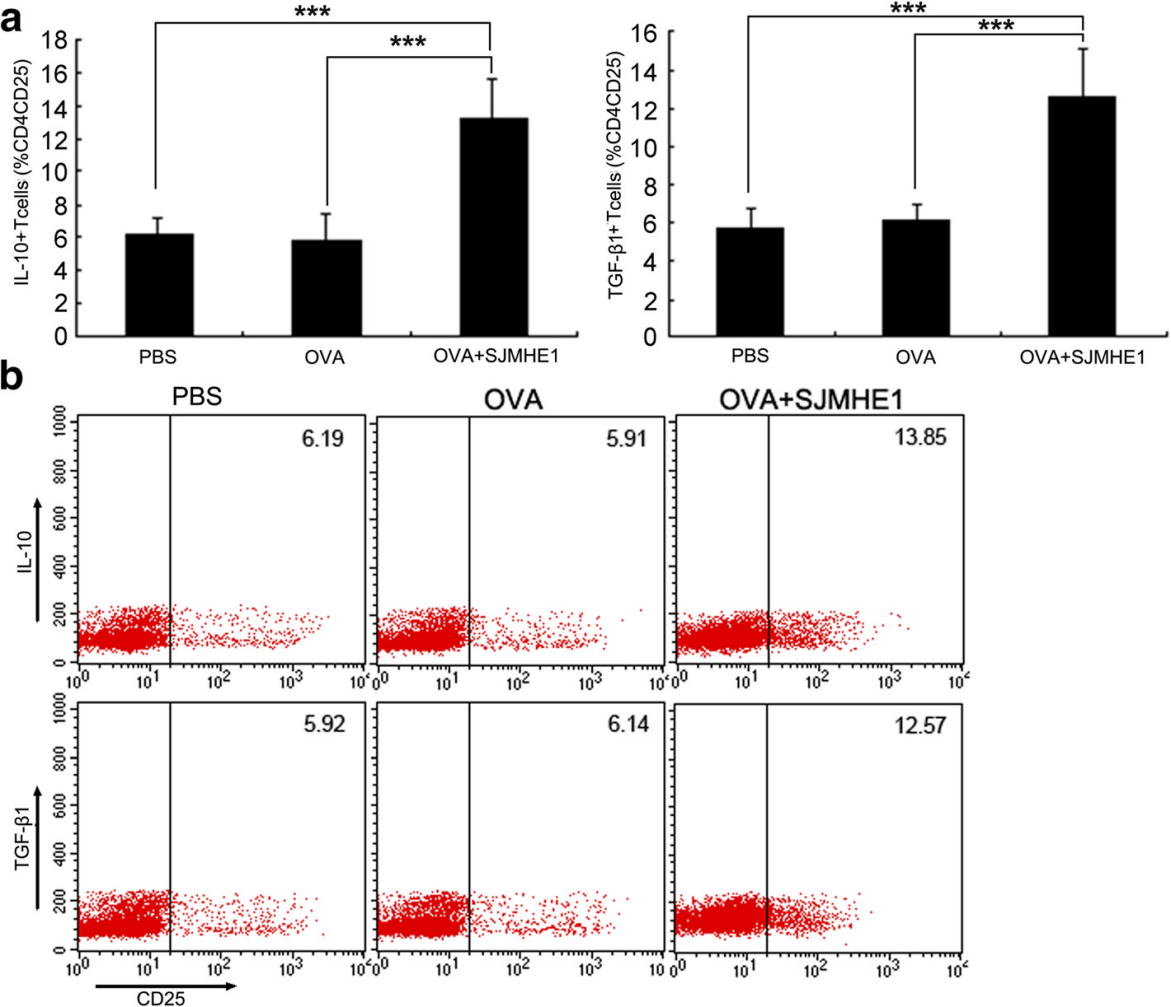
SJMHE1 induced IL-10 and TGF-β1 expression in CD4^+^CD25^+^ T-cells during DTH. **a** BALB/c mice were sensitised with OVA alone or combined with 30 μg of SJMHE1. Challenge with OVA occurred 7 days later; 24 h after the challenge, spleen and LNs from each mouse were pooled. Single-cell suspensions were prepared, and red blood cells were lysed. Intracellular expression levels of IL-10 and TGF-β1 were analysed by flow cytometry. Events were gated on CD3, CD4, and CD25 expression as indicated. Values indicate the percentage of events in the indicated quadrant. Data are expressed as the mean ± SD of 18 mice from three independent experiments; **b** Expression of intracellular IL-10 and TGF-β1 in gated CD4^+^CD25^+^ cells was analysed by flow cytometry. Data are representative of the experiments. Asterisks indicate significant differences analysed using the independent Student’s *t*-test (***P < 0.001)

Splenic lymphocytes from mice immunised with OVA combined with SJMHE1 produced high levels of anti-inflammatory cytokines (IL-10 and TGF-β1). Thus, we determined the relationship of the upregulated IL-10 and TGF-β1 in SJMHE1-treated mice with the SJMHE1-induced CD4^+^CD25^+^ T-cells. We further investigated the expression of intracellular IL-10 and TGF-β1 in the CD4^+^CD25^+^ T-cells from mice sensitised with OVA alone or combined with SJMHE1. Flow cytometric analysis revealed higher expression levels of intracellular IL-10 (*t* = 11.18, *P* < 0.001) and TGF-β1 (*t* = 10.10, *P* < 0.001) in the CD4^+^CD25^+^ T-cells from SJMHE1-immunised mice than in those from OVA-injected mice (Fig. 4). These results indicate that the production of IL-10 and TGF-β1 by CD4^+^CD25^+^ T-cells contributes to SJMHE1-mediated inhibition.

### IL-10 and TGF-β1 mediated the inhibition of the proliferation of responder T-cells from DTH mice by SJMHE1-induced CD4^+^CD25^+^ Tregs

**Fig. 5:**
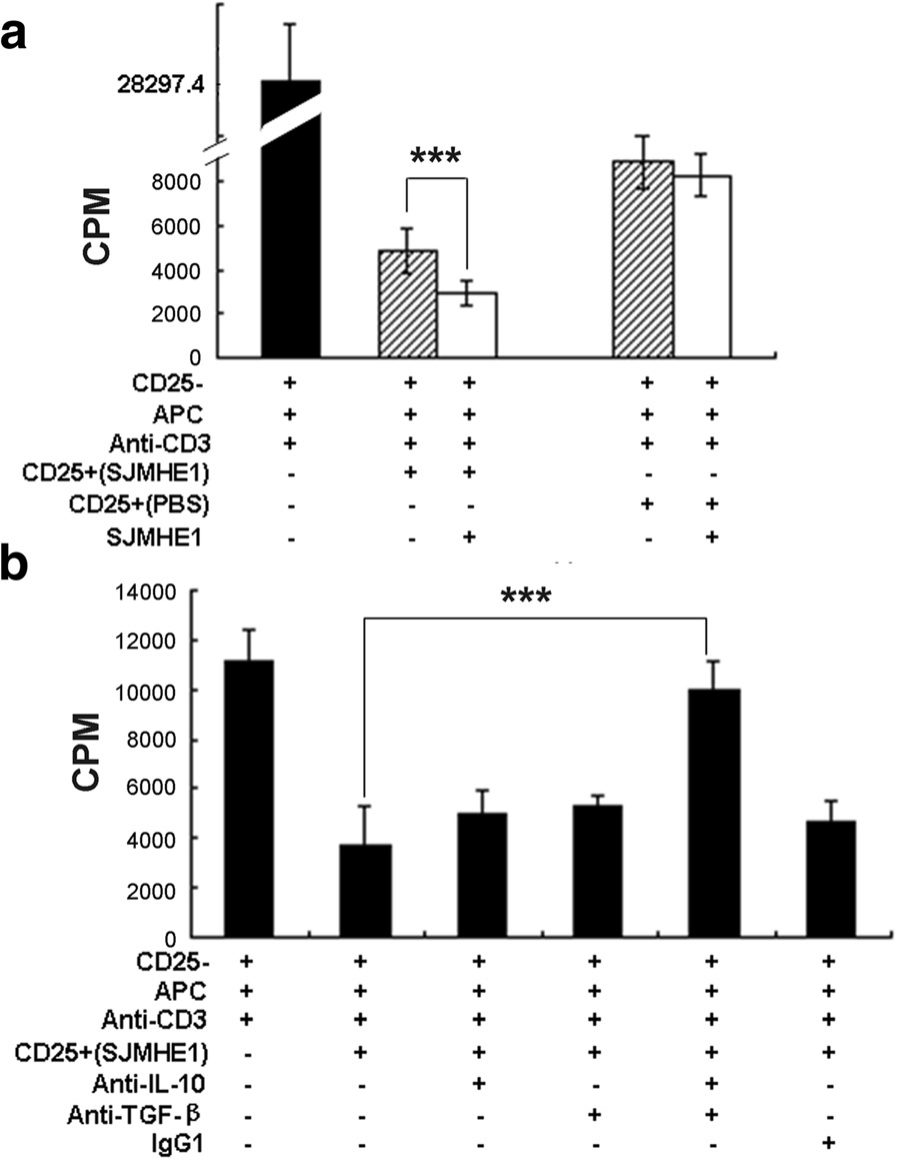
IL-10 and TGF-β1 mediated the inhibition of the proliferation of responder T-cells from DTH mice by SJMHE1-induced CD4^+^CD25^+^ Tregs. **a** Responder CD4^+^CD25^−^ T-cells (1 × 10^5^ cells/well) and irradiated APCs (1 × 10^5^ cells/well) from DTH mice (primed and challenged with OVA alone) were cultured with CD4^+^CD25^+^ T-cells (5 × 10^4^ cells/well) harvested from either SJMHE1- or PBS-treated mice and stimulated with anti-CD3 (1 μg/mL) in the presence or absence of SJMHE1 (0.1 μg/mL); **b** CD4^+^CD25^−^ T-cells (1 × 10^5^ cells/well) and irradiated APCs (1 × 10^5^ cells/well) from DTH mice were cultured for 72 h either alone or with CD4^+^CD25^+^ T-cells (5 × 10^4^ cells/well) from SJMHE1-immunised mice and stimulated with anti-CD3 (1 μg/mL). Wells contained either anti-IL-10 (3 μg/mL), anti-TGF-β1 (0.5 μg/mL), both antibodies, or isotype control antibodies. Proliferation was measured by ^3^H-thymidine incorporation for the last 16 h of the experiment. Data are shown as the mean ± SD (*n* = 6 per group) from three independent experiments. Asterisks indicate significant differences analysed using the independent Student’s *t*-test (***P < 0.001)

We used CD4^+^CD25^+^ cells from either SJMHE1- or PBS-treated mice to assess further the suppressive efficacy of SJMHE1-induced CD4^+^CD25^+^ Tregs. Each of the two groups of enriched CD4^+^CD25^+^ cells was co-incubated with the CD4^+^CD25^−^ T-cells from mice primed and challenged with OVA alone (established DTH mice). As shown in Fig. 5a, the CD4^+^CD25^+^ T-cells from the two immunised mouse groups were highly effective in suppressing CD4^+^CD25^−^ T-cell proliferation after stimulation with anti-CD3 Ab. However, the CD4^+^CD25^+^ T-cells purified from SJMHE1-immunised mice induced the highest inhibition. Compared with the CD4^+^CD25^+^ T-cells purified from PBS-immunised mice, the CD4^+^CD25^+^ T-cells generated from SJMHE1-immunised mice showed significantly enhanced inhibitory ability after the addition of SJMHE1 to co-cultures (*t* = 7.232, *P* < 0.001).

Considering that the SJMHE1-induced CD4^+^CD25^+^ T-cells secreted both IL-10 and TGF-β1, we tested whether or not these cytokines mediate the suppressor function of CD4^+^CD25^+^ T-cells *ex vivo*. The CD4^+^CD25^−^ T-cells from the mice primed and challenged with OVA alone (established DTH mice) were co-incubated with SJMHE1-induced CD4^+^CD25^+^ T-cells with or without anti-IL-10, anti-TGF-β1 neutralizing mAb, or a mixture of anti-IL-10, anti-TGF-β1, or their IgG1 isotype controls. As shown in Fig. 5b, the *ex vivo* suppressive activities of CD4^+^CD25^+^ T-cells were partially reversed by the addition of anti-IL-10 or anti-TGF-β1 mAb to the culture medium. This property demonstrates that the SJMHE1-induced CD4^+^CD25^+^ T-cells partly mediate their suppressive effects via IL-10 or TGF-β1. However, the mixture of anti-IL-10 and anti-TGF-β1 mAb completely blocked the suppressive activity mediated by CD4^+^CD25^+^ T-cells (*t* = 14.05, *P* < 0.001). These results suggest that both IL-10 and TGF-β1 mediate the inhibition of CD4^+^CD25^+^ T-cells induced by SJMHE1 during DTH.

### SJMHE1 induced the generation of peripheral CD4^+^CD25^+^ Tregs from CD4^+^CD25^−^ T-cells

**Fig. 6:**
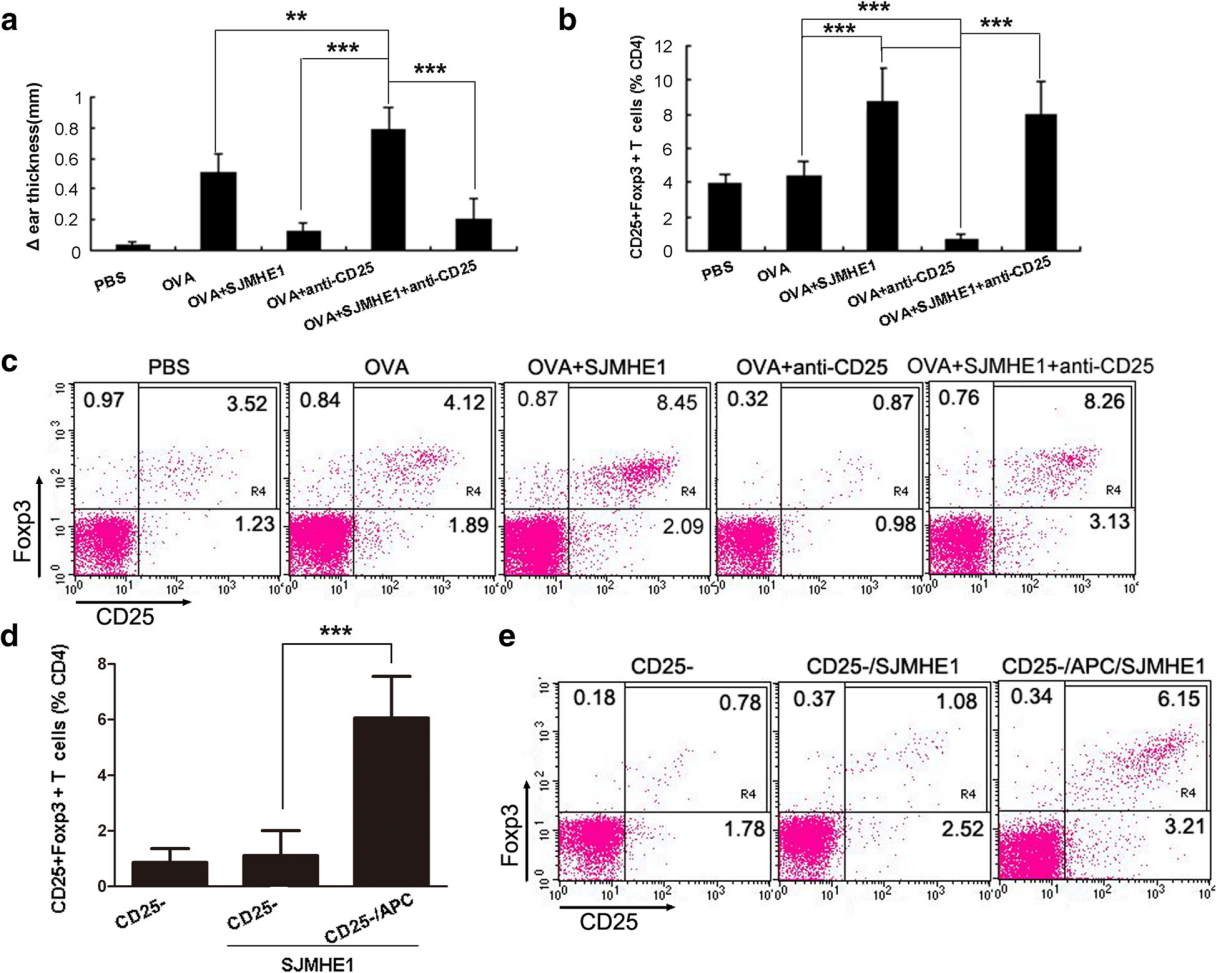
SJMHE1 induced the generation of peripheral CD4^+^CD25^+^ Tregs from CD4^+^CD25^−^ T-cells. BALB/c mice were injected with anti-CD25 Ab to eliminate CD25^+^ T-cells and sensitised with OVA alone or combined with 30 μg of SJMHE1 after 24 h. **a** DTH responses were assessed over the subsequent 24 h with the change in ear thickness. DTH responses are expressed as the mean ± SD of 12 mice from two independent experiments; **b** Challenge with OVA occurred 7 days later; 24 h after the challenge, spleen and LNs from each mouse were pooled. FCM analysis for CD3, CD4, CD25, and Foxp3 was performed, and data are expressed as the mean ± SD of 18 mice from three independent experiments; **c** One representative experiment of the total data shown in **b**; **d** Splenic CD4^+^CD25^−^ T-cells (2 × 10^5^/well) isolated from DTH mice (primed and challenged with OVA alone) were stimulated with 0.1 μg/mL SJMHE1 in the absence or presence of APC (2 × 10^5^/well) for 3 days. The cells were stained with PerCP-anti-CD3, FITC-anti-CD4, APC-anti-CD25, and PE-anti-Foxp3 and then examined by flow cytometry. Data are expressed as the mean ± SD of two independent experiments (n = 6 per group); **e** Representative experiment of the total data shown in **d**. Asterisks indicate significant differences analysed using the independent Student’s *t*-test. (**P < 0.01; ***P < 0.001)

CD4^+^CD25^+^ Tregs can be generated peripherally from CD4^+^CD25^−^ T-cells [31]. We performed depletion experiments of CD4^+^CD25^+^ T-cells to determine whether or not the SJMHE1-induced increase in CD4^+^CD25^+^ Tregs during DTH is attributable to the expansion of the existing naturally occurring CD4^+^CD25^+^ Tregs or to newly generated Tregs from CD4^+^CD25^−^ T-cells. The mice were injected with anti-mouse CD25 mAb, and the depletion of CD25-expressing cells was confirmed using FACS. After 24 h, the mice were primed and challenged to induce DTH. We tested the CD4^+^CD25^+^FoxP3^+^ T-cells from mice in each group 24 h after the challenge. As shown in Fig. 6a, the depletion of CD25^+^ T-cells prior to OVA immunisation enhanced the severity of DTH responses as compared with the mice immunised with OVA alone or combined SJMHE1. The proportion of CD4^+^CD25^+^Foxp3^+^ T-cells significantly increased in the spleens and LN of the SJMHE1-administered mice than in those of the OVA-immunised (*t* = 8.486, *P* < 0.001) or PBS-treated mice (*t* = 9.709, *P* < 0.001) regardless of the depletion of CD25^+^ T-cells (Fig. 6b and c). CD25^+^ depletion exerted no influence on the beneficial effect of SJMHE1. The mice depleted of CD25^+^ T-cells and immunised with OVA and SJMHE1 possessed almost the same number of spleen and lymph CD4^+^CD25^+^Foxp3^+^ T-cells as the SJMHE1-treated undepleted mice (Fig. 6b and c). The results suggest that SJMHE1 induces the generation of peripheral CD4^+^CD25^+^ Tregs from CD4^+^CD25^−^ T-cells. To confirm this hypothesis, CD4^+^CD25^−^ T-cells isolated from the spleen and LNs of DTH mice were stimulated *ex vivo* with SJMHE1 in the absence or presence of APCs. As shown in Fig. 6d and e, the incubation with SJMHE1 significantly increased the percentage of CD4^+^CD25^+^Foxp3^+^ T-cells in an APC-dependent manner (*t* = 9.802, *P* < 0.001). This result suggests that SJMHE1 induces the peripheral generation of CD4^+^CD25^+^Foxp3^+^ T-cells from the CD4^+^CD25^−^ compartment.

## Discussion

Parasite such as *Schistosoma mansoni* have co-evolved with the immune systems of their mammalian hosts; thus, they have established a strong regulatory and anti-inflammatory network to ensure their safety inside these hosts [19, 32–34]. Schistosome infection modulates the progression of autoimmune diseases, such as experimental colitis [12], experimental allergic encephalomyelitis [35], Graves’ disease [36], and type 1 diabetes [37]. Thus, considerable interest has been drawn toward defining the molecules derived from schistosomes, which can replace live infection to prevent or control pro-inflammatory pathological responses [19]. Furthermore, identifying and characterising immunomodulatory molecules from various pathogens is an expanding area of research that should provide an opportunity to uncover many natural inflammation modulators with the potential for use as novel immunotherapeutics to treat immune-mediated human diseases [15, 16].

In that regard, some parasite-derived immunomodulators from helminths have recently been reported [15]. The most well-defined nematode-derived immunomodulatory molecule to date is ES-62, a phosphorylcholine-containing glycoprotein secreted by the rodent filarial nematode *Acanthocheilonema viteae.* This molecule has well-conserved orthologs in human filarial nematode parasites, including *Brugia malayi* and *Onchocerca volvulus* [13, 17, 38, 39]. ES-62 exhibits a wide range of anti-inflammatory properties [40–42]; thus, the molecule has been tested in mouse models of both autoimmune and allergic diseases and has been reported to protect against collagen-induced arthritis [42, 43] and type I hypersensitivity in the skin and lungs [44].

The most important finding of the current study is that the administration of the parasite-derived immunomodulatory molecule SJMHE1 from *S. japonicum* can inhibit DTH responses in a mouse model. SJMHE1 is composed of overlapping T-cell epitopes and is highly identical to murine and human HSP60. Consistent with the previous observation, the “share epitope” cross-recognition of auto-reactive T-cells reportedly protects against autoimmune and inflammatory disorders in experimental animal models [45, 46]. The combination of OVA and SJMHE1 more greatly suppressed DTH responses to a single OVA challenge compared with OVA alone. The attenuation of inflammation in DTH mice by SJMHE1 treatment was associated with a reduction in pro-inflammatory cytokines (TNF-α and IL-12) and a concomitant increase in anti-inflammatory cytokine production (IL-10 and TGF-β1) by inflammatory sites and T-cells. Increased IL-10 and TGF-β1 production from both the local inflammatory sites and by *ex vivo* splenic T-cells indicate that SJMHE1-stimulated immunomodulatory responses occur both in local and systemic tissues. These results are consistent with other parasite products, such as body fluid from *Ascaris suum* (ABF), which suppresses DTH responses in mice by co-immunisation with OVA at the time of priming. This suppression was partially mediated by the anti-inflammatory cytokine IL-10 [47].

CD4^+^CD25^+^ Tregs are essential for the maintenance of peripheral tolerance and the control of the immune response. Consistent with SJMHE1 treatment leading to the increase in the population of CD4^+^CD25^+^Foxp3^+^ T-cells [24], SJMHE1 induced the generation and/or activation of CD4^+^CD25^+^ Tregs during DTH; this phenomenon suppressed the inflammatory response in DTH mice. In addition to expanding the CD4^+^CD25^+^ Treg population, SJMHE1 also induced Tregs efficient in both cytokine secretion and suppressive activity. A characteristic marker of Tregs is the constitutive expression of CTLA-4, a negative regulatory factor critical for the induction and function of Tregs [48, 49]. Consistent with these reports, SJMHE1-induced CD4^+^CD25^+^ Tregs expressed high levels of CTLA-4, explaining the partial dependence of the regulatory activity of these cells. SJMHE1-induced CD4^+^CD25^+^ Tregs also produced high levels of IL-10 and TGF-β1, which significantly contribute to the suppressive properties of CD4^+^CD25^+^ T-cells *ex vivo*. The mechanisms involved in the generation/activation of Tregs by SJMHE1 during DTH are not fully understood. However, the present study showed that they can be peripherally generated from CD4^+^CD25^−^ T-cells because SJMHE1 administration inhibited DTH response in CD25-depleted mice and restored the number of CD4^+^CD25^+^ Tregs. In addition, SJMHE1 induced the *ex vivo* generation of CD4^+^CD25^+^Foxp3^+^ T-cells from activated CD4^+^CD25^−^ T-cells of DTH mice in an APC-dependent manner. These data are consistent with our previous finding that SJMHE1 induces the differentiation of tolerogenic DCs and MΦs with the capacity to generate CD4^+^CD25^+^ Tregs *in vitro* [24]. Therefore, we hypothesised that SJMHE1 can generate CD4^+^CD25^+^ Tregs from the peripheral CD4^+^CD25^−^ compartment by inducing tolerogenic APCs and augment IL-10 and TGF-β production in DTH mice. The production of IL-10 and TGF-β might further promote the development of Tregs [19]. The cooperation between Tregs and the anti-inflammatory cytokines IL-10 and TGF-β1 would contribute to the therapeutic effect of SJMHE1 on autoimmune and inflammation disorders. Furthermore, these “safe” selective generated anti-inflammatory signals, which have evolved during host-parasite interactions, can be used to provide unique tools for defining key molecular events in the development of an anti-inflammatory response and for defining new therapeutic targets [50].

Considerable effort has recently been directed toward the enhancement or restoration of Treg functions for therapeutic immunointervention in autoimmune and inflammatory diseases. Therapeutic restoration or boosting of the Treg compartment *in vivo* by small-molecule or biopharmaceutical therapeutics would allow for such a treatment to be more affordable and more widely available than customised Treg therapy. In favor of such a strategy, several experimental models have demonstrated that many immunosuppressive peptides could elicit Treg development in the periphery and protect against autoimmune diseases, such as collagen-induced arthritis [51], myasthenia gravis [52], and multiple sclerosis [53]. The inhibition of DTH responses by SJMHE1 in the current study is consistent with previous results indicating that the active suppression by other peptides is mediated by the induction of CD4^+^CD25^+^ Tregs, the downregulation of inflammatory cytokines, and the upregulation of IL-10 and TGF-β1 secretion [54, 55]. The potential use of SJMHE1 as a therapeutic peptide for the treatment of allergic and autoimmune diseases requires further analysis.

## Conclusions

The HSP60 peptide SJMHE1 derived from *S. japonicum* can effectively inhibit DTH. SJMHE1 suppresses pro-inflammatory cytokines, enhances anti-inflammatory cytokine production by the cells in both the local tissues and the immune system, and generates CD4^+^CD25^+^ Tregs that depend on the production of IL-10 and TGF-β1 to suppress DTH responses. Thus, SJMHE1 possessing immunomodulatory properties can have potential therapeutic applications for the treatment of inflammatory disorders.

## Abbreviations

APCs: antigen presenting cells
DTH: delayed type hypersensitivity
FCM: flow cytometry
HSP60: Heat shock protein 60
OVA: ovalbumin
PBS: phosphate buffer solution
s.c.: subcutaneously
Tregs: regulatory T-cells
